# Partial Characterization of Biosurfactant from *Lactobacillus pentosus* and Comparison with Sodium Dodecyl Sulphate for the Bioremediation of Hydrocarbon Contaminated Soil

**DOI:** 10.1155/2013/961842

**Published:** 2013-04-15

**Authors:** A. B. Moldes, R. Paradelo, X. Vecino, J. M. Cruz, E. Gudiña, L. Rodrigues, J. A. Teixeira, J. M. Domínguez, M. T. Barral

**Affiliations:** ^1^Chemical Engineering Department, School of Industrial Engineering (EEI), University of Vigo, Campus As Lagoas, Marcosende, 36310 Vigo-Pontevedra, Spain; ^2^Edaphology and Agricultural Chemistry Department, Facultad de Farmacia, University of Santiago de Compostela, Campus Universitario Sur. 15782, Santiago de Compostela, La Coruña, Spain; ^3^Institute for Biotechnology and Bioengineering (IBB), Centre of Biological Engineering, University of Minho, Campus de Gualtar, 4710-057 Braga, Portugal

## Abstract

The capability of a cell bound biosurfactant produced by *Lactobacillus pentosus*, to accelerate the bioremediation of a hydrocarbon-contaminated soil, was compared with a synthetic anionic surfactant (sodium dodecyl sulphate SDS-). The biosurfactant produced by the bacteria was analyzed by Fourier transform infrared spectroscopy (FTIR) that clearly indicates the presence of OH and NH groups, C=O stretching of carbonyl groups and NH nebding (peptide linkage), as well as CH_2_–CH_3_ and C–O stretching, with similar FTIR spectra than other biosurfactants obtained from lactic acid bacteria. After the characterization of biosurfactant by FTIR, soil contaminated with 7,000 mg Kg^−1^ of octane was treated with biosurfactant from *L. pentosus* or SDS. Treatment of soil for 15 days with the biosurfactant produced by *L. pentosus* led to a 65.1% reduction in the hydrocarbon concentration, whereas SDS reduced the octane concentration to 37.2% compared with a 2.2% reduction in the soil contaminated with octane in absence of biosurfactant used as control. Besides, after 30 days of incubation soil with SDS or biosurfactant gave percentages of bioremediation around 90% in both cases. Thus, it can be concluded that biosurfactant produced by *L. pentosus* accelerates the bioremediation of octane-contaminated soil by improving the solubilisation of octane in the water phase of soil, achieving even better results than those reached with SDS after 15-day treatment.

## 1. Introduction 

The extensive production and use of hydrocarbons have resulted in widespread environmental contamination by these chemicals. Contaminated sites must be cleaned because of the toxicity and persistence of these compounds and the associated negative effects on living organisms. Hydrocarbons, which are hydrophobic organic compounds, are poorly soluble in groundwater and tend to partition to the soil matrix. The partitioning can account for as much as 90–95% or more of the total contaminant mass. As a consequence, hydrocarbon contaminants are moderately to poorly recovered by physic-chemical treatments, display limited bioavailability to microorganisms and limited availability to oxidative and reductive chemicals when applied *in situ* and/or *ex situ* [1].

On the other hand, lignocellulose residues like the pruning waste generated in vineyards are usually burnt in the field, releasing greenhouse gases and cancerous compounds such as polycyclic aromatic hydrocarbons. Therefore, the use of vineyard pruning waste as a carbon source in biosurfactant production could decrease the environmental impact associated with burning this type of waste in the field [2, 3]. 

Bustos et al. [3] showed that *Lactobacillus pentosus* produces lactic acid and biosurfactants by utilizing hemicellulosic sugars in vineyard pruning waste, and Portilla et al. [2] demonstrated that *Lactobacillus acidophilus* produces biosurfactants by utilizing the cellulosic fraction of vineyard pruning waste. Moreover, Moldes et al. [4] produced biosurfactants using *L. pentosus* grown on synthetic media, composed by glucose and xylose and they used the biosurfactant obtained for the bioremediation of octane contaminated soil. These authors found that biosurfactant from *L. pentosus*, grown on synthetic media, reduced the concentration of octane in the soil to 58.6% and 62.8%, for soil charged with 700 and 70,000 mg Kg^−1^ of hydrocarbon, respectively. However, there are no comparative studies about the use of biosurfactants produced by *L. pentosus* and chemical surfactants, such as sodium dodecyl sulfate (SDS). 

Moreover, it is important to point out that although biosurfactant from *L. pentosus* has been proposed, in previous works [4], as surfactant for the bioremediation of contaminated soil, in the literature there are no studies about the composition of this biosurfactant. Thus, thinking about the further application and commercialisation of this biosurfactant it would be interesting to elucidate its composition.

In the current work, in order to know the composition of the biosurfactant produced by *L. pentosus* during the fermentation of hemicellulosic sugars, which can be obtained from vineyard pruning waste, this was analysed by Fourier transform infrared spectroscopy (FTIR) and the corresponding spectrum was compared with those obtained for other biosurfactants, also produced by lactic acid bacteria. Moreover, soil samples contaminated with octane were treated with the biosurfactant produced by *L. pentosus* or with SDS and incubated for several days, in order to test the comparative efficacy of the two types of surfactants in the bioremediation of hydrocarbon-contaminated soil. 

## 2. Materials and Methods

### 2.1. Microorganism

*Lactobacillus pentosus* CECT-4023 T (ATCC-8041) was obtained from the Spanish Collection of Type Cultures (Valencia, Spain). The strain was grown on the complete media proposed by Mercier et al. [5], at 31°C for 24 h. Inocula were prepared by solubilisation of cells from plates, with 5 mL of sterilized hemicellulosic sugars.

### 2.2. Biotechnological Production of Bisurfactant

Hemicellulosic sugars containing 18 g/L xylose, 10.6 g/L glucose, and 3.9 g/L arabinose, that could be obtained by acid hydrolysis of vineyard pruning waste at 130°C with 3% sulphuric acid for 15 minutes using a liquid/solid ratio of 8 g/g [2, 3]; were supplemented with 10 g L^−1^ of yeast extract (YE) and 10 g L^−1^ of corn steep liquor (CSL) and used directly as fermentation media. The chemostat fermentation was carried out in a 2 L Applikon fermentor at 250 rpm, with a working volume of 1.4 L, at 31°C and with pH adjusted to 5.85. 

### 2.3. Extraction of Biosurfactant

The bacterial cells were recovered by centrifugation, washed twice in demineralized water, and resuspended in 50 mL of phosphate-buffer saline (PBS: 10 mM KH_2_PO_4_/K_2_HPO_4_ and 150 mM NaCL, pH adjusted to 7.0). The suspensions were maintained at room temperature for up to 2 hours, with gentle stirring to encourage release of biosurfactants. The bacteria were removed from the solution by centrifugation, and the remaining supernatant liquid (containing the biosurfactants) was filtered through a 0.22 *μ*m pore-size filter (Millipore) for analysis and evaluation. 

### 2.4. Surface Activity Determination

The surface activity of the biosurfactant was determined by measuring the surface tension of the samples with the ring method. The surface tension of the PBS extract containing the biosurfactants produced by *L. pentosus* was measured using a KRUSS K6 Tensiometer was equipped with a 1.9 cm Du Noüy platinum ring. To increase the accuracy of the measurements, the measurements were made in triplicate.

The concentration of biosurfactant (mg L^−1^) was determined from a calibration curve: concentration (mg L^−1^) = (surface tension (mN/m) − 76.9)/−8.65 [6]. The calibration curve was calculated for a commercial biosurfactant produced by several bacilli (surfactin). Surfactin is a biosurfactant produced by a Bacillus strain, similar to *Lactobacillus pentosus* and it is commercially available. Different concentrations of biosurfactant solution were tested, below the critical micelle concentration (CMC) of known surface tension. The decrease in surface tension is linear in this range of concentration and it is therefore possible to establish a relationship between the concentration of biosurfactant and the surface tension [7]. Nevertheless, in order to estimate the concentration of biosurfactant, the solution was diluted to the CMC.

### 2.5. Fourier Transform Infrared Spectroscopy

Fourier transform infrared spectroscopy (FTIR) is particularly useful for identifying different types of chemical bonds (functional groups) and can therefore be used to identify the components of mixtures of unknown composition. Molecular characterization was performed with a crude biosurfactant mixture extract, which was dialyzed against demineralized water at 4°C in a Spectrapor membrane tube (molecular weight cut off 6000–8000, Spectrum Medical Industries Inc., CA, USA), and then freeze-dried. One milligram of freeze-dried crude biosurfactant was ground with 100 mg of KBr and with a pressure of 7500 kg was applied for 30 s in order to produce translucent pellets, which were then analysed by spectrometry (FT/IR-4200, JASCO). All spectra were obtained from 180 scans with a resolution of 4 cm^−1^ in the range of 550–4000 cm^−1^. A KBr pellet was used as background reference. Moreover, previously to FTIR analysis total soluble protein and reducing sugars content of biosurfactant was analysed by Lowry and the phenol sulphuric method, respectively.

### 2.6. Relative Emulsion Volume and Stability of the Biosurfactant Produced by *L. pentosus*

Two mL of octane was mixed with an equal volume of the PBS containing the biosurfactant produced by *L. pentosus*, or with sodium dodecyl sulfate (SDS) at its critical micelle concentration (CMC), following the protocol published by Portilla et al. [2]. The hydrocarbons and surfactants were mixed and shaken vigorously for 2 min., and left for 1 h. The relative emulsion volume (EV, %) and stability (ES, %) were measured at this time (i.e., at time 0 h), and 24 h later, and the EV and ES were calculated from (1), proposed by Das et al. [8] as follows:

(1) $$\begin{array}{c} \text{EV},\%=\frac{\text{Emulsion}\;\;\text{height},\text{mm}\times \text{Cross}\;\;\text{section}\;\;\text{area},{\text{mm}}^{2}}{\text{Total}\;\;\text{liquid}\;\;\text{volume},{\text{mm}}^{3}},\\ \%\text{ES}=\frac{\%\text{EV},\;\;\text{at}\;\;\text{time}\;\;\text{h}}{\text{EV},\;\;\text{at}\;\;0\;\text{h}}*100. \end{array}$$

### 2.7. Soil Samples

The soil samples were sieved (2 mm) prior to analysis. The water content was estimated by drying the soil at 105°C until constant weight, as described by Guitián and Carballas [9]. The pH was determined either in water or 0.1 N KCl, at a soil:solution ratio of 1 : 1.5, and measured after 10 min and 2 hours, respectively. Total organic carbon (TOC) and organic matter (OM) were determined by oxidation with a mixture of K_2_Cr_2_O_7_ and H_2_SO_4_ and titration with Mohr Salt, following the method proposed by Guitián and Carballas [9]. The particle size distribution (coarse sand, 2–0.2 mm; fine sand, 0.2–0.05 mm; coarse silt, 0.05–0.02 mm; fine silt, 0.02–0.002 mm, and clay <0.002 mm) was determined by the Robinson pipette method, after wet sieving, as described by Guitián and Carballas [9]. The nitrogen (N) content was determined by wet digestion with H_2_SO_4_, using the Kjeldhal method as described by Guitián and Carballas [9]. Dehydrogenase activity (DHA) was measured by the reduction of 2,3,5-triphenyltetrazolium chloride (TTC) to triphenyl formazan (TPF), following the method described by Tabatabai [10]. The octane content of the soil was analysed in triplicate, by headspace gas chromatography.  Table 1 shows the soil composition assayed in this work.

### 2.8. Incubation Experiments

The soil was contaminated up to 7,000 mg Kg^−1^ of octane and then incubated in the presence and absence of sodium dodecyl sulfate (SDS) or the biosurfactant produced by *L. pentosus*. The octane concentration selected, for contaminating soil, is in the range of those used in a previous work [4]. The surfactant/soil ratio was 1 : 5 (liquid : solid), and surfactants were added to soil, in Erlenmeyer flasks, at the CMC. The flasks were then incubated at 25°C for 30 days, without shaking. In order to study the effect of the biosurfactant on the bioremediation of octane, soil contaminated with octane in absence of biosurfactant was included in the set of experiments as a control. Moreover, in order to evaluate the effect of microbial activity on the bioremediation of hydrocarbon-contaminated soil, samples of soil were contaminated with 7,000 mg Kg^−1^ of octane and sterilized. The octane concentration of soil was analyzed in triplicate by headspace gas chromatographic.

## 3. Results and Discussion

### 3.1. Characterization of Biosurfactants Produced by *L. pentosus*, by Fourier Transform Infrared Spectroscopy

**Figure 1:**
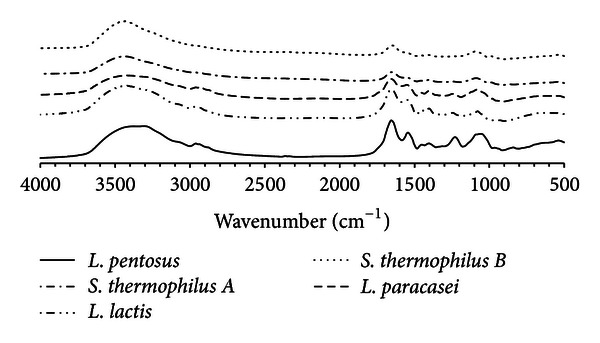
FTIR spectra of biosurfactant produced by *L. pentosus* in comparison with the spectra obtained for biosurfactants produced by other lactic acid bacteria (*L. lactis*, *L. pentosus*, and* S. thermophilus A* and *B*).

The FTIR method has been widely used to characterize the surface groups, since infrared (IR) transmission spectra present peaks characteristic of specific chemical bonds [11]. Comparison of the peaks and corresponding chemical groups for the biosurfactant produced by *L. pentosus* and biosurfactants produced by other lactic acid bacteria (*Lactococcus lactis*, *Lactobacillus paracasei*, *Streptococcus thermophilus A*, and *Streptococcus thermophilus B*) are shown in Figure 1 and Table 2 [12–14]. The presence of a 3200–3500 cm^−1^ peak in the *L. pentosus* biosurfactant spectrum clearly indicates the presence of OH and NH groups in glycoproteins, structures proposed for the biosurfactants produced by *L. lactis* and *L. paracasei* [6, 14, 15]. A peak at 1725 and 1675 cm^−1^ corresponding to C=O stretching of carbonyl groups and NH bending (peptide linkage) was also observed in the spectrum obtained for the *L. pentosus* biosurfactant. Furthermore, important peaks were also observed at 2900 cm^−1^ (CH_2_–CH_3_ stretching) and at 1000–1200 cm^−1^ (C–O stretching in sugars). A glycolipid-like structure has previously been proposed for the biosurfactants produced by strains of *S. thermophilus* [12, 13], although some characteristic protein/peptide groups were observed in the FTIR spectra. Comparison of the spectrum obtained for the *L. pentosus* biosurfactant with those reported for the other biosurfactants revealed that the *L. pentosus* biosurfactant is more closely related to those produced by *L. lactis* and *L. paracasei*, suggesting that could be a glycoprotein or a glycolipopeptide (Figure 1).

Moreover it was found that the bisurfactant from *L. pentosus* was composed by 44.7 ± 1.5% soluble protein and 13.4 ± 2.9% total sugars that confirm the results found in the FTIR analysis, although in the future it will be necessary to determine the lipid content in order to clarify if biosurfactant from *L. pentosus* is a glycoprotein or a glycolipopeptide.

### 3.2. Study of the Emulsifying Capacity of Biosurfactants Produced by *L. pentosus*

Biosurfactants have often been used to enhance the bioavailability and biodegradation of hydrophobic compounds, but knowledge of the effect of biosurfactants on the biodegradation of complex hydrocarbon mixtures is limited [1]. In previous works [16] the emulsifying capacity of bisourfactants produced by *L. pentosus* was evaluated and it was found that when this strain is grown on sugars from agricultural residues, it produces biosurfactants with emulsifying properties, which could facilitate the bioremediation of hydrocarbon-contaminated sites. 

In the present study, the capacity of biosurfactants produced by *L. pentosus* to stabilize emulsions octane/water was evaluated; it was found that the biosurfactants obtained after growing this strain on hemicellulosic sugars that can be obtained by vineyard pruning waste, yielded a relative emulsion volume (EV) of about 40.0%, and 85.7% stability (ES) after 24 h. The relative emulsion volume (EV) and stabilizing capacity value (ES) for emulsions of octane/water stabilized by biosurfactants produced by *L. pentosus* or SDS, in comparison with the results reported in previous works [16] are shown in Table 3. The capacity of the biosurfactant produced in the present study to stabilize octane/water emulsions is similar to that of the biosurfactants assessed in previous works [16], which were produced by the same strain (although grown on a different culture media), to stabilize gasoline/water emulsions. In previous works [16] it was found that among the biosurfactants assayed, those produced by bacteria grown with distilled grape marc hydrolyzate as a substrate yielded the highest EV (45.5%) after 24 h, followed by the biosurfactants produced with hazelnut as a substrate. The EV values were higher than those reported for commercial surfactin (22.3% for gasoline and 30.4% for kerosene) and lower than those reported for SDS (66.2% for gasoline and 62.3% for kerosene). The same was observed in the present study for the *L*. *pentosus* biosurfactant and SDS when used to stabilize octane/water emulsions. 

### 3.3. Bioremediation of Hydrocarbon-Contaminated Soil

The physicochemical characteristics of the soil assayed in the present study are shown in Table 1. The soil comprised 69.7% sand and 20.7% clay, and the pH was 5. The organic matter content was 11.2 g/Kg, the total nitrogen concentration, 0.9 g Kg^−1^ with a C/N ratio about 12.4. The dehydrogenase activity (DHA) was approximately 334 mg TPF Kg^−1^, accounting for the microbial activity of the soil. 

On the other hand, the surfactants tested in the present study for the bioremediation of hydrocarbon-contaminated soil were sodium dodecyl sulfate and the biosurfactant produced by *L. pentosus*. Sodium dodecyl sulfate (SDS or NaDS), or sodium lauryl sulfate (SLS) (C_12_H_25_SO_4_Na) is an anionic surfactant used in many cleaning and hygiene products. The CMC of SDS is about 0.0082 M in pure water at 25°C. SDS was applied to the soil at its CMC (0.0082 M in pure water). The biosurfactant was obtained by the fermentation of hemicellulosic sugars (18 g/L xylose; 10.6 g/L glucose and 3.9 g/L arabinose) that can be obtained by hydrolysis of vineyard pruning waste. The biosurfactant produced by *L. pentosus* reduced the surface tension of PBS from 72 mN m^−1^ to 54 mN m^−1^, and this was diluted to its CMC (2.65 mg L^−1^) before being added to the soil samples. 

Regarding the utilization of surfactants for the bioremediation of hydrocarbon-contaminated soil, Urum et al. [17] investigated the efficiency of different surfactant solutions to remove crude oil from contaminated soils, by a soil washing process. The authors demonstrated that the synthetic surfactant-sodium dodecyl sulphate (SDS) and rhamnolipid biosurfactants were more efficient at removing the crude oil than natural surfactants saponins. However, no studies have compared the ability of SDS and biosurfactants produced by lactic acid bacteria to biodegrade hydrocarbons in soil.

**Figure 2:**
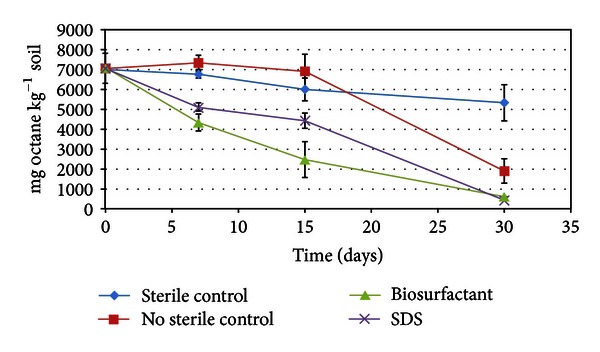
Kinetic profile of the biodegradation of octane in soil, in the presence and absence of surfactants (SDS or biosurfactant produced by *L. pentosus*).

In this work soil was contaminated up to 7,000 mg Kg^−1^ of octane. After 15 days of treatment, the contaminated soil reduced the octane concentration by 65.1% and 37.2% when was treated with the biosurfactants produced by *L. pentosus* or the SDS, respectively, whereas in the untreated soil, consisting of soil contaminated with octane in absence of biosurfactant or surfactant, the octane concentration was only reduced by about 2.2% (Figure 2). However the greatest reduction in the octane concentration was observed after 30 days of incubation (92 and 94% for soil containing biosurfactant or SDS, resp.). Figure 2 shows the kinetic profile of octane biodegradation in the soil after 30 days of treatment, in presence and absence of biosurfactant. Regarding the effectiveness of the biosurfactant, the greatest differences in the biodegradation of octane were achieved after 15 days of treatment. After this time, the octane concentration remained at around 6,000 and 6,900 mg Kg^−1^ in untreated soils (sterilized and unsterilized soil, resp.), whereas in the treated soil samples, the octane concentration was reduced to 2,469 and 4,400 mg Kg^−1^ by the *L. pentosus* bisurfactant and SDS, respectively. However, after 30 days, the octane concentration decreased to 591 mg Kg^−1^ and 430 mg Kg^−1^ in samples incubated with the *L. pentosus* bisurfactant and SDS, respectively. 

These data demonstrate that biosurfactant accelerates the solubilisation of octane in water, improving the degradation of this contaminant by the microbial biomass of soil. Moreover, in Figure 2 it can be observed that, in the sterilized soil in absence of biosurfactant, the octane concentration remains almost stable during 30 days of treatment in comparison with the nonsterilized control (nonsterilized soil in absence of biosurfactant). This fact can be explained on the basis that in nonsterilized soil exists microbial biomass that after 15 days of adaptation to the medium is able to metabolize the octane contained in the soil, whereas in the sterilized soil there are no microbial biomasses that can metabolize this hydrocarbon. In this case the advantage of using biosurfactant from *L. pentosus* for the bioremediation of octane contaminated soil focuses on the ability of the biosurfactant to accelerate the biodegradation process in presence of microbial biomass.

SDS is one of the most typical surfactants proposed in the literature for the bioremediation of hydrocarbon-contaminated soil [18, 19]; for example, it has been demonstrated that enhances desorption and biodegradation of phenanthrene in soil-water systems. It is not carcinogenic when applied directly to skin or consumed (CIR 1983) [20]. However, it has been shown to irritate facial skin after prolonged and constant exposure (more than an hour) in young adults [21]. Thus it is interesting to look for more friendly environmental surface-active compounds.

In comparison with SDS, biosurfactants produced by *L. pentosus* could be considered nontoxic since they are produced by a generally regarded as safe (GRAS) microorganism. In fact, lactic acid bacteria, most of which are biosurfactant producers, are widely consumed in food products. It can therefore be speculated that these cell-bound biosurfactants would not be toxic to humans or animals, and therefore could be used in many applications such as the bioremediation of contaminated sites, in place of other chemical surfactants.

## 4. Conclusion

Owing to their biodegradability, low toxicity and effectiveness, biosurfactant produced by *L. pentosus* is a very promising compound for use in the bioremediation of contaminated soil, because it was able to increase the solubilisation of octane in the aqueous-soil systems and thus improved its biodegradation, showing even higher capability for the bioremediation of hydrocarbon-contaminated soil than SDS. Moreover, from the FTIR analysis it can be concluded that biosurfactant from *L. pentosus* could be a glycoprotein or a glycolipopeptide and the use of hemicellulosic sugars from inexpensive substrates (such as vineyard pruning waste) may enable the large-scale production of biosurfactants. 

## Figures and Tables

**Table 1:** Physicochemical characterization of the soil assayed in the present study.

Properties	Units	Value
pH_H_2_O_		5.1
pH_KCl_		4.0
Sand	%	69.7
Coarse Silt	%	3.0
Fine Silt	%	6.6
Clay	%	20.7
Texture		Loam-clayey-sandy
TOC (Total organic carbon)	g/Kg	11.2
N	g/Kg	0.9
C/N		12.4
OM	g/Kg	19.3
Octane	mg/Kg	185
DHA (Dehydrogenase activity)	mg·TPF/kg·day	334

**Table 2:** Correspondence between IR spectra and functional groups detected in the biosurfactant produced by *L. pentosus* and in biosurfactants produced by other lactic acid bacteria (*L. lactis*, *L. paracasei*, and *S. thermophilus A* and *B*).

Absorbance band (cm^−1^)	Region
3200–3600	OH and NH stretching
2900–2950	C–H (stretching) groups CH_2_ and CH_3_
1725, 1675	C=O (stretching)
1520	N–H bending in proteins
1400–1460	C–H bending vibrations of CH_3_ and CH_2_ groups; CH (scissor)
1100–1090	OH deformation vibrations/CN
1000–1300	C–O sugar stretching

**Table 3:** Comparison of relative emulsion volume (EV) and stability (ES) after 24 h, of octane/water emulsions formed and stabilized with the biosurfactant produced by *L. pentosus* during fermentation of sugars in vineyard pruning waste and sodium dodecyl sulfate (SDS). Data are compared with EV and ES values reported for gasoline or kerosene and surfactin, SDS or biosurfactants produced by *L. pentosus*.

Hydrocarbon	EV (%)	ES (%)	Surfactant	Reference
Gasoline	38.9–45.5	85.0–94.7	Biosurfactant produced by *L. pentosus* with lignocellulosic residues as substrate	[16]
Gasoline	22.3	64.6	Surfactin produced by *Bacillus subtilis *	[16]
Gasoline	66.2	96.1	SDS	[16]

Kerosene	21.7–49.0	84.9–99.0	Biosurfactant produced by *L. pentosus* with lignocellulosic residues as substrate	[16]
Kerosene	30.4	73.1	Surfactin produced by *Bacillus subtilis *	[16]
Kerosene	62.3	87.7	SDS	[16]

Octane	39.8	85.7	Biosurfactant produced by *L. pentosus* using hemicellulosic sugars that could be obtained from trimming vineyard.	Present study
Octane	64.0	94.0	SDS	Present study
